# Draft genome sequences of multiple *Salmonella enterica* serotypes isolated from eight different animals in Nigeria

**DOI:** 10.1128/mra.00204-25

**Published:** 2025-07-02

**Authors:** Ibrahim A. Raufu, Opeyemi U. Lawal, Valeria R. Parreira, Mitra Soni, Harmanpreet Kaur, Akeem O. Ahmed, Abdulfatai Aremu, Ahmad I. Al-Mustapha, Lawrence Goodridge

**Affiliations:** 1 Department of Veterinary Microbiology, University of Ilorin 108285, Ilorin, Nigeria; 2 Canadian Research Institute for Food Safety, University of Guelph 3653, Guelph, Ontario, Canada; 3 The Global Bacterial Antimicrobial Resistance Sequencing (GLOBAR-SEQ) Program, Guelph, Ontario, Canada; 4 School of the Environment, University of Windsor 468155, Windsor, Ontario, Canada; 5 Department of Veterinary Pharmacology and Toxicology, University of Ilorin 108285, Ilorin, Nigeria; 6 Department of Food Hygiene and Environmental Health, Faculty of Veterinary Medicine, University of Helsinki 124453 , Helsinki, Finland; 7 Department of Veterinary Services, Kwara State Ministry of Agriculture and Rural Development, Ilorin, Kwara, Nigeria; University of Maryland School of Medicine, Baltimore, Maryland, USA

**Keywords:** *Salmonella*, Zoonotic pathogens, one health, food safety

## Abstract

*Salmonella enterica* is a zoonotic pathogen that poses a significant threat to public health. It is the leading cause of bacterial foodborne illness with complex transmission dynamics across human, animal, and environmental pathways. Here, we report draft genomes of 25 *S*. *enterica* isolates recovered from different animals.

## ANNOUNCEMENT

*Salmonella enterica* is a zoonotic pathogen that poses a significant threat to public health (1, 2). It is highly diverse and comprises over 2,600 serotypes, many of which are implicated in foodborne illnesses globally (1, 3). The One Health approach is crucial for understanding the epidemiology, environmental reservoirs, and the multifactorial drivers of *S. enterica* (2, 4, 5). Here we present the draft genomes of 25 *S*. *enterica* isolates recovered from eight animal species. These isolates are a subset of bacterial whole-genome sequencing (WGS) data in the Global Bacterial Antibiotic Resistance Sequencing (GLOBAR-SEQ) program collection (6, 7).

Fecal and intestinal samples were collected from slaughterhouse processing in Ilorin, Nigeria, as part of routine surveillance and were exempt from ethical approval. Ten grams of each was enriched in 90 mL of buffered peptone water (Fluka BioChemika, Germany) and incubated at 35℃ for 20 hours. Subsequently, 1 mL was inoculated into 9 mL of Selenite-F broth (Fluka BioChemika, Germany) and incubated at 35℃ for 20 hours. Cultures were transferred onto *Salmonella-Shigella* agar (Laboratorios Britania, Argentina) and incubated at 35℃ overnight. Black colonies were streaked on xylose-lysine-desoxycholate (XLD) agar (Oxoid, UK) and incubated at 35℃ for 20 hours. Presumptive *Salmonella* isolates were shipped to the Canadian Research Institute for Food Safety for sequencing. Pure colonies were characterized as *Salmonella* using the VITEK system (BioMérieux, Canada). Genomic DNA was extracted using the DNEasy Blood and Tissue kit (Qiagen, Germany). Libraries were prepared using the Illumina DNA prep Tagmentation kit, and paired end (2 × 150 bp) sequencing was performed on the Illumina NextSeq 1000 system (6).

Sequencing of 25 *Salmonella* isolates produced a median of 1,698,429 paired-end reads. Raw reads were quality filtered using FastQC v0.11.9 (https://github.com/s-andrews/FastQC), trimmed with Trimmomatic v0.39 (8), and assembled using SKESA v2.4.0 (9). Assembly quality was assessed using QUAST v5.2 (10). Genome completeness was assessed using CheckM2 v1.1.0 (11). *In silico* serotyping of isolates was determined using SISTR v1.1.2 (12). Genomes were annotated using the NCBI Prokaryotic Genome Annotation Pipeline v6.9 (13). Antimicrobial resistance genes were identified using the CARD v4.0.0 database (14), and species classification was confirmed as *S. enterica* using average nucleotide identity with JSpeciesWS (15). Default settings were used for all bioinformatics tools.

On average, the draft genomes produced 29 contigs, guanine-cytosine (GC) content of 52.19%, an N50 of 469,406, and a genome size of 4,586,339 bp, with coverage from 69 to 129× and 100% genome completeness. The genomes contained an average of 4,362 protein-coding sequences, 72 tRNAs, and 123 pseudogenes. Seven distinct serotypes were identified in the collection, with Poona (*n* = 8) and Panama (*n* = 7) being the most prevalent. Additionally, rare serotypes Soerenga and Weltevreden, which are infrequently reported in the region, were detected. Three isolates from chicken and cattle belonging to two serotypes harbored the plasmid-mediated *qnrB5* gene for quinolone resistance (16). Another isolate, N_SE_190 (serotype Albany), from a chicken spleen, carried resistance genes for sulfonamides (*sul1*), tetracycline (*tet(A*)), and biocides (*qacEΔ1*) (Table 1). Overall, these genomes will enrich available data for *S. enterica* to advance One Health approaches for enhanced surveillance of zoonotic pathogen.

**TABLE 1:** Summary of sequence metrics of the 25 *Salmonella enterica* isolates recovered from multiple animals

ID	City, country	Year	Isolation source	Specimen type	Host	% GC	# Read	# Contigs	Genome size (bp)	% Genome completeness	Coverage	N50	Serovar	# tRNAs	# Coding sequence	# Pseudogenes	# AMR genes	SRA	GenBank
N_SE_189	Ilorin, Nigeria	2019	Poultry	Liver	Poultry	52.2	1920067	19	4528839	100	112.54	685776	Give	74	4296	124	*aac(6')-Iy*	SRR32115514	JBLVHI000000000
N_SE_190	Ilorin, Nigeria	2019	Poultry	Spleen	Poultry	52.2	1941878	23	4778585	100	104.69	773636	Albany	75	4558	134	*aac (3)-Id, aac(6')-Iaa, aph(3")-Ib, aph(3')-Ia, aph (6)-Id, aadA7, qacEdelta1, sul1, tet(A*)	SRR32115515	JBLVHJ000000000
N_SE_235	Ilorin, Nigeria	2019	Poultry	Cecum	Poultry	52.2	1229651	21	4528872	100	72.06	610117	Give	74	4297	126	*aac(6')-Iy*	SRR32115503	JBLVHH000000000
N_SE_237	Ilorin, Nigeria	2019	Poultry	Cecum	Poultry	52.2	1240683	22	4528663	100	73.32	618490	Give	73	4299	123	*aac(6')-Iy*	SRR32115497	JBLVHG000000000
N_SE_249	Ilorin, Nigeria	2019	Poultry	Spleen	Poultry	52.2	1214074	17	4555527	100	72.78	598882	Give	73	4321	123	*aac(6')-Iy, qnrB5*	SRR32115496	JBLVHF000000000
N_SE_267	Ilorin, Nigeria	2019	Poultry	Spleen	Poultry	52.2	1827269	29	4568874	100	110.47	392019	Montevideo	72	4348	112	*aac(6')-Iaa, qnrB5*	SRR32115495	JBLVHE000000000
N_SE_311	Ilorin, Nigeria	2018	Poultry	Cecum	Poultry	52.2	1870229	36	4566507	100	110.01	391179	Montevideo	75	4345	110	*aac(6')-Iaa, qnrB5*	SRR32115494	JBLVHD000000000
N_SE_332	Ilorin, Nigeria	2021	Avian section	Feces	Captive wild bird	52	1766265	38	4951953	100	97.76	359437	Soerenga	75	4734	140	*aac(6')-Iy*	SRR32115500	JBLVGN000000000
N_SE_337	Ilorin, Nigeria	2021	Horse stable	Feces	Horse	52.1	1638872	22	4472084	100	102.3	651369	Panama	70	4230	124	*aac(6')-Iy*	SRR32115513	JBLVGZ000000000
N_SE_340	Ilorin, Nigeria	2021	Hypro feeds	Feed	Poultry	52.3	1733787	32	4538075	100	104.1	393292	Poona	71	4313	115	*aac(6')-Iaa*	SRR32115501	JBLVGO000000000
N_SE_341	Ilorin, Nigeria	2021	Horse stable	Feces	Captive horses	52.3	1593607	30	4540341	100	96.36	385317	Poona	70	4314	116	*aac(6')-Iaa*	SRR32115509	JBLVGV000000000
N_SE_344	Ilorin, Nigeria	2021	Horse stable	Feces	Horse	52.1	1964671	21	4472074	100	120.07	548236	Panama	72	4227	124	*aac(6')-Iy*	SRR32115510	JBLVGW000000000
N_SE_345	Ilorin, Nigeria	2021	Horse stable	Feces	Horse	52.3	1887241	32	4539646	100	114.46	385266	Poona	74	4317	117	*aac(6')-Iaa*	SRR32115504	JBLVGQ000000000
N_SE_346	Ilorin, Nigeria	2022	Fish market	Skin	Fish	52.1	1169075	20	4470156	100	72.91	726643	Panama	70	4229	125	*aac(6')-Iy*	SRR32115491	JBLVHA000000000
N_SE_347	Ilorin, Nigeria	2021	Horse stable	Feces	Horse	52.1	1103315	25	4470862	100	68.78	481466	Panama	70	4228	124	*aac(6')-Iy*	SRR32115512	JBLVGY000000000
N_SE_352	Ilorin, Nigeria	2021	Carnivores section	Feces	Captive Hyena	52.1	1882733	24	4470997	100	116.21	393029	Panama	62	4229	124	*aac(6')-Iy*	SRR32115493	JBLVHC000000000
N_SE_354	Ilorin, Nigeria	2021	Avian section	Feces	Captive wild bird	52.3	1698429	31	4539526	100	103.32	390746	Poona	74	4316	116	*aac(6')-Iaa*	SRR32115508	JBLVGU000000000
N_SE_357	Ilorin, Nigeria	2022	Fish market	Intestine	Fish	52.3	1495057	27	4539532	100	88.98	398725	Poona	73	4311	117	*aac(6')-Iaa*	SRR32115506	JBLVGS000000000
N_SE_358	Ilorin, Nigeria	2022	Fish market	Intestine	Fish	52.3	1821631	29	4539754	100	106.39	476495	Poona	74	4312	115	*aac(6')-Iaa*	SRR32115505	JBLVGR000000000
N_SE_365	Ilorin, Nigeria	2021	Avian section	Feces	Captive wild bird	52.3	2135530	31	4541242	100	128.45	406646	Poona	73	4316	117	*aac(6')-Iaa*	SRR32115507	JBLVGT000000000
N_SE_368	Ilorin, Nigeria	2021	Horse stable	Feces	Horse	52.3	1523986	26	4538210	100	90.55	406646	Poona	73	4315	116	*aac(6')-Iaa*	SRR32115502	JBLVGP000000000
N_SE_372	Ilorin, Nigeria	2021	Avian section	Feces	Captive wild bird	52.1	1652579	23	4471753	100	102.35	340992	Panama	65	4229	124	*aac(6')-Iy*	SRR32115492	JBLVHB000000000
N_SE_376	Ilorin, Nigeria	2021	Horse stable	Feces	Horse	52.1	1598740	68	5016983	100	88.79	175460	Weltevreden	69	4881	148	*aac(6')-Iy*	SRR32115499	JBLVGM000000000
N_SE_377	Ilorin, Nigeria	2021	Horse stable	Feces	Horse	52.1	1890845	21	4469654	100	115.7	607872	Panama	68	4226	124	*aac(6')-Iy*	SRR32115511	JBLVGX000000000
N_SE_380	Ilorin, Nigeria	2021	Horse stable	Feces	Horse	52.1	1556539	66	5019766	100	85.48	137424	Weltevreden	70	4878	147	*aac(6')-Iy*	SRR32115498	JBLVGL000000000

## Data Availability

This Whole-Genome Shotgun project has been deposited at DDBJ/ENA/GenBank under the accession BioProject accession number PRJNA1215413. SRA and assemblies’ accessions are listed in Table 1.
